# Positive thyroid transcription factor 1 staining strongly correlates with survival of patients with adenocarcinoma of the lung

**DOI:** 10.1038/sj.bjc.6602717

**Published:** 2005-07-26

**Authors:** F Barlési, D Pinot, A LeGoffic, C Doddoli, B Chetaille, J-P Torre, P Astoul

**Affiliations:** 1 Faculty of Medicine – Assistance Publique Hôpitaux de Marseille, Department of Thoracic Oncology, Fédération des Maladies Respiratoires, Hôpital Sainte-Marguerite, 13274 Marseille Cedex 09, France; 2 Faculty of Medicine – Assistance Publique Hôpitaux de Marseille, Department of Pathology, Hôpital Sainte-Marguerite, 13274 Marseille Cedex 09, France; 3 Assistance Publique Hôpitaux de Marseille, Department of Thoracic Surgery, Hôpital Sainte-Marguerite, 13274 Marseille Cedex 09, France; 4 Assistance Publique Hôpitaux de Marseille, Department of Medical Information, Hôpital de la Timone, 13285 Marseille Cedex 05, France

**Keywords:** non-small-cell lung cancer, adenocarcinoma, thyroid transcription factor 1, prognosis, differentiation, metastatic stage

## Abstract

This study investigated the relation between positive thyroid transcription factor 1 (TTF1) staining and survival of patients affected by primary adenocarcinoma (ADC) of the lung. Pathological tissue from consecutive ADC patients was collected from 2002 to 2004. The anti-TTF1 antibody (8G7G3/1, dilution of 1/200) was used. Thyroid transcription factor 1 staining was assessed for each tumour as positive or negative. Probability of survival was estimated by Kaplan–Meier and difference tested by log-rank test. A Cox's regression multivariate analysis was carried out. In all, 106 patients were studied (66% male, 69% PS0–1, 83% with stage III or IV). Tumours expressed positive TTF1 staining in 66% of cases. Multivariate analysis demonstrated an independent lower risk of death for patients whose tumour expresses positive TTF1 staining (HR=0.51, 95% CI 0.30–0.85; *P*=0.01) and higher grade of differentiation (HR=0.40, 95% CI 0.24–0.68; *P*=0.001). In conclusion, positive TTF1 staining strongly and independently correlates with survival of patients with primary ADC of the lung.

Primary adenocarcinoma (ADC) of the lung has risen in incidence, now reaching approximately 50% of non-small-cell lung cancer (NSCLC). However, identification of primary ADC is sometimes difficult but has been enhanced by the use of the thyroid transcription factor 1 (TTF1) immunostaining. Indeed, positive TTF1 staining has been shown for 75–80% of primary ADC while negative for virtually all squamous carcinoma (SCC) of the lung as well as for extrapulmonary but thyroid tumours (Stenhouse *et al*, 2004).

The prognostic value for a positive TTF1 staining has been associated with contradictory results for NSCLC patients. A study on 222 stage I NSCLC patients demonstrated no survival difference associated with TTF1 immunoreactivity (Pelosi *et al*, 2001), while another study correlated a positive TTF1 staining of resected NSCLC with a poor survival (Puglisi *et al*, 1999). On the other hand, four studies on early stages of NSCLC associated a positive TTF1 staining with a longer survival (Haque *et al*, 2002; Myong, 2003; Tan *et al*, 2003; Saad *et al*, 2004). However, all these studies were interested in surgical NSCLC patients and all but one (Saad *et al*, 2004) included both ADC and non-ADC histological types of NSCLC.

The aim of the present study was to investigate the relation of a positive TTF1 staining with survival of patients (1) affected exclusively by a primary ADC of the lung and (2) mainly presenting with locally advanced or metastatic stage of the disease.

## PATIENTS AND METHODS

All consecutive patients diagnosed with ADC from January 2002 to May 2004 at our department were included in the study. Histological subclassification was carried out according to the World Health Organization classification (Travis *et al*, 1999). Performance status (PS) was estimated using the Eastern Cooperative Oncology Group (ECOG) scale. Clinical examination and computed tomographic scan of the chest, abdomen and brain were carried out systematically.

**Figure 1:**
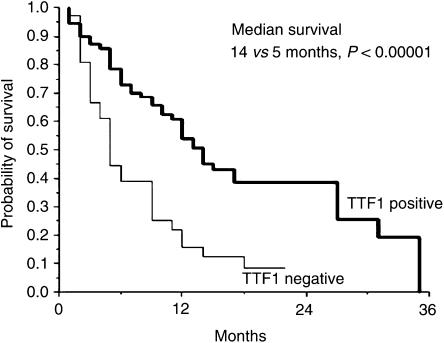
Survival curves of patients whose tumours showed positive and negative TTF1 staining.

Pathological tissue was obtained from each patient before treatment. The pathological tissue was obtained either from the primary tumour in the majority of cases, or from mediastinal lymph nodes (*n*=10) or distant metastases (*n*=5). The pathological tissue was extracted from surgical specimens when available. Tumours have been classified into poorly, moderately and well-differentiated tumours (Travis *et al*, 1999). Immunohistochemical staining was carried out on formalin-fixed, paraffin-embedded tissue samples, using a standard streptavidin–biotin-based method. The anti-TTF1 antibody (8G7G3/1, mouse monoclonal antibody; Dako, Ely, Cambridgeshire, UK) was used at a dilution of 1/200. Scoring was carried out independently by two pathologists (AL and BC). For each tumour, neoplastic cells were assessed as positive or negative for TTF1 staining (Figure 1). Sole nuclear staining was considered as a positive result. Any positive nuclear staining was sufficient to deem a tumour as positive for TTF1 staining. In each case, normal alveolar walls served as positive internal control. All patients consented to treatment and were treated with thoracic surgery, radiation therapy and/or chemotherapy, in accordance with national and international guidelines (Depierre *et al*, 2003) after a multidisciplinary assessment of their disease.

Survival data were updated in March 2005. One patient was lost to follow-up. Probability of survival was estimated using the Kaplan–Meier method. Differences between survival were tested by means of log-rank test. A multivariate regression analysis was carried out with Cox's regression using the forward maximum likelihood method. All variables with a *P*-value less than 0.20 at the time of univariate analysis were entered into the model. A *P*-value less than 0.05 was considered as significant.

## RESULTS

In all, 106 patients were included into the study (Table 1). Tumours expressing positive TTF1 staining were associated neither with demographics (i.e. gender and age) nor with disease characteristics (i.e. PS, TNM stage or presence of metastasis). Thyroid transcription factor 1 staining was not associated with tumours differentiation, as poorly, moderately and well-differentiated tumours expressed a positive TTF1 staining in 21 (30% of the tumours showing positive TTF1 staining), 35 (50%) and 14 (20%) cases, respectively (*P*=0.177).

**Figure 2:**
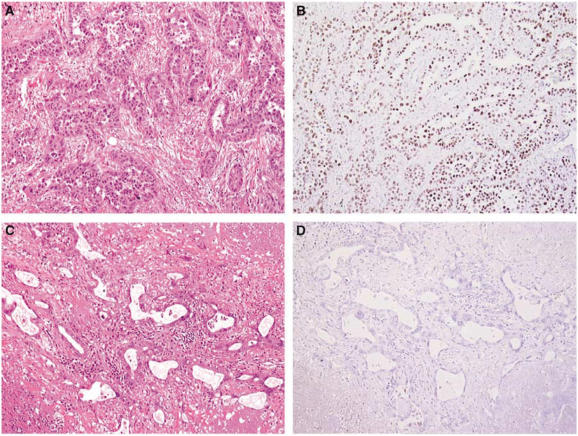
Acinar ADC (haematoxylin and eosin, × 100) (**A**) showing positive nuclear TTF1 staining (**B**), and acinar ADC (haematoxylin and eosin, × 100) (**C**) showing negative TTF1 staining (**D**).

At the time of analysis, 75 patients were deceased. In univariate analysis, tumours differentiation and TTF1 staining significantly correlated with survival. Tumours stage as well as PS also influenced survival, without reaching statistical significance (Table 2). Multivariate analysis demonstrated a statistically significant and independent lower risk of death for patients whose tumour expresses a positive TTF1 staining (HR=0.40, 95% CI 0.25–0.65; *P*<0.0001) (Figure 2), and a statistically significant and independent lower risk of death for patients whose tumour expresses higher grade of differentiation (HR=0.64, 95% CI 0.46–0.91; *P*=0.01).

## DISCUSSION

This study highlights the decreased risk of death for patients with primary ADC of the lung showing a positive TTF1 staining (HR=0.40, 95% CI 0.25–0.65; *P*<0.0001).

Previous studies suggested either a favourable or an unfavourable prognosis for patients with tumour showing a positive TTF1 staining. However, all these studies but one (Saad *et al*, 2004) included NSCLC patients with a mixture of histological types. However, the prevalence of a positive TTF1 staining varies throughout histological types, from 80% for ADC to less than 10% for SCC. This heterogeneity probably explains the conflicting results previously reported. In addition, available data on the prognosis associated with TTF1 staining considered exclusively surgically treated NSCLC patients. Thus, the results reported herein extend the favourable prognosis related to a positive TTF1 staining to patients with advanced stage of primary ADC of the lung.

The reasons why TTF1 is related to prognosis of patients with primary ADC of the lung are unclear. A positive TTF1 staining has been inversely related to the proliferative activity evaluated through Ki-67 expression, usually considered as a marker of poor prognosis in NSCLC (Pelosi *et al*, 2001; Myong, 2003). On the other hand, a correlation between positive TTF1 staining and various molecular markers expression such as p53 or HER2/neu involved in lung carcinogenesis has been suggested (Haque *et al*, 2002; Yatabe *et al*, 2002; Myong, 2003). Thus, further biological investigations are needed.

Finally, a significant relationship between positive TTF1 staining and female gender or nonsmoker status has been suggested (Yatabe *et al*, 2002). Thus, a correlation with epidermal growth factor receptor tyrosine kinase inhibitors activity should be prospectively investigated. In fact, a TTF1 influence on patients' survival through difference in treatment sensibility might be hypothesised.

In conclusion, positive TTF1 staining strongly and independently correlates with survival of patients with ADC of the lung by a way to be identified.

## Figures and Tables

**Table 1:** Main clinical and pathological characteristics of the 106 patients

	***n* (%)**
Age, <70/⩾70 years	82 (77)/24 (23)
Gender, women/men	36 (34)/70 (66)
PS, 0–1/⩾2 (ECOG)	73 (69)/33 (31)
*Stage* a	
Early stage (I and II)	18 (17)
Locally advanced stage (IIIA and IIIB)	35 (33)
Distant metastasis (IV)	53 (50)
*Histological differentiation*	
Poorly differentiated	38 (36)
Moderately differentiated	47 (44)
Well differentiated	21 (20)
TTF1 staining (positive/negative)	70 (66)/36 (33)

a UICC Classification.

PS=performance status; TTF1=thyroid transcription factor 1.

**Table 2:** Major results of univariate analysis

	**Median survival (months)**	***P***
*PS*		
0 or 1	12	NS
⩾2	5	
*Stage*		
I and II	10	NS
IIIA and IIIB	12	
IV	7	
*TTF1 staining*		
Positive	14	<0.00001
Negative	5	
*Differentiation*		
Poorly	5	0.004
Moderately	13	
Well	12	

PS=performance status (ECOG); TTF1=thyroid transcription factor 1; NS=nonsignificant.
